# Bullying and Cyberbullying in Childhood and Adolescence: A Narrative Review and Proposed Clinical Response Framework

**DOI:** 10.3390/healthcare14193180

**Published:** 2026-09-25

**Authors:** Ghada Shahrour, Fatma M. Ibrahim

**Affiliations:** 1 Faculty of Nursing, Jordan University of Science and Technology, Irbid 22110, Jordan; 2 RAK College of Nursing, RAK Medical and Health Sciences University, Ras Al Khaimah 11172, United Arab Emirates; fatma_magdi@mans.edu.eg; 3 Faculty of Nursing, Mansoura University, Mansoura 35516, Egypt

**Keywords:** bullying, cyberbullying, child, adolescent, mental health

## Abstract

Background: Existing reviews establish that bullying and cyberbullying are associated with substantial health burden, but they rarely integrate conceptual distinctions, evidence strength, and healthcare response. Objective: To synthesize evidence on traditional bullying, peer victimization, and cyberbullying among children and adolescents and to identify clinically defensible implications and research priorities. Methods: A structured narrative review informed by SANRA addressed three questions concerning construct boundaries, health consequences and modifiers, and healthcare response. PubMed/MEDLINE was searched through 29 August 2026; Google Scholar supported citation tracing and the retrieval of named institutional reports. Eligibility, selection, charting, and narrative evidence-characterization procedures are reported. The final synthesis included 61 sources (53 journal articles and eight institutional reports, guidance documents, or books). Results: Cross-national surveillance estimated 11% repeated school bullying and 15% cybervictimization under different frequency thresholds. Meta-analytic estimates were 25% for victimization and 16% each for perpetration and bully–victim involvement. A longitudinal meta-analysis of 27 studies (13,497 participants) found a small prospective association between cybervictimization and later mental-health symptoms. School programs reduced perpetration by approximately 18–19% and victimization by 15–16%; cyberbullying effects were small (g = −0.18 and −0.13, respectively). The evidence was strongest for associations with internalizing symptoms, suicidality, psychosomatic complaints, sleep disruption, and impaired functioning but bidirectionality and residual confounding limit causal inference. Conclusions: Symptom- or risk-triggered, behavior-specific inquiry and coordinated safety assessment are justified; evidence is insufficient to recommend universal clinical screening. BV-CARE is presented as an author-developed, hypothesis-generating framework requiring stakeholder co-design, feasibility testing, and prospective validation before routine use.

## 1. Introduction

Bullying-related harm may move across classrooms, transport, neighborhoods, group chats, gaming spaces, and social-media platforms. In healthcare, disclosure is often indirect: recurrent pain, sleep disturbance, school avoidance, unexplained injury, anxiety, deteriorating functioning, aggression, or self-harm may precede any explicit report of peer aggression. This clinical presentation makes precise definitions and proportionate response important.

The Centers for Disease Control and Prevention defines bullying as unwanted aggressive behavior by another youth or group of youths that involves an observed or perceived power imbalance and repetition or a high likelihood of repetition [1]. These criteria distinguish bullying from conflict between peers of similar power [1,2]. Peer victimization is broader and may include harmful peer acts without documented repetition or power imbalance. Cyberbullying is digitally mediated aggression in which repetition and power can be produced through forwarding, persistent content, audience reach, anonymity, or control of private material [3,4]. Because instruments operationalize these constructs differently, their prevalence estimates are not automatically comparable.

Definitions also determine clinical scope. A one-time assault, sexual-image threat, or identity-based incident may not satisfy a research definition of bullying but can still require urgent care or safeguarding. Conversely, labeling every reciprocal disagreement as bullying can obscure the assessment of power and pattern. This review therefore preserves the constructs as related but non-identical and uses behavior, impact, power, and recurrence to guide interpretation.

Bullying is a public-health and child-rights concern [5,6]. In the 2021–2022 Health Behaviour in School-aged Children survey, 11% reported school bullying at least two or three times per month during the previous couple of months, whereas 15% reported cybervictimization at least once or twice during that period [7]. These different thresholds must remain visible. A 2025 meta-analysis of 116 studies involving more than 600,000 participants estimated pooled prevalences of 25% for victimization and 16% each for perpetration and bully–victim involvement, with substantial heterogeneity [8].

Health evidence is most consistent for internalizing symptoms, suicidality, psychosomatic complaints, sleep disturbance, and impaired functioning [9,10,11,12,13,14,15,16,17,18,19]. However, much of this evidence is observational, and longitudinal findings support reciprocal relationships rather than a simple one-way causal model [10,11,17]. Neurodevelopmental conditions, chronic illness, and minority stress may increase exposure or modify consequences [20,21,22,23,24,25,26,27], but effect estimates vary by population and measurement. These qualifications preclude deterministic or single-cause interpretations and support individualized, risk-stratified assessment [5,28,29].

The existing reviews typically examine prevalence, traditional bullying, cyberbullying, selected health outcomes, or school interventions separately [3,4,30]. They provide limited integration of construct-specific measurement, comparative evidentiary strength, symptom-triggered clinical recognition, and the boundary between evidence-supported care and expert-derived practice proposals. This structured narrative review addresses that translational gap. It asks the following questions: (1) How are bullying, peer victimization, and cyberbullying related yet operationally distinct? (2) Which health consequences, mechanisms, modifiers, and intervention effects are supported by the strongest evidence? (3) Which healthcare responses are supported, plausible but untested, or inappropriate to recommend without further evaluation?

## 2. Materials and Methods

### 2.1. Review Design and Objectives

This structured narrative review was informed by SANRA [31]. Its interpretive purpose was to integrate heterogeneous evidence and translate it cautiously for healthcare; it was not designed to estimate pooled effects, claim exhaustive retrieval, or substitute for a systematic review. The three review questions stated in the Introduction defined the evaluation aim and scope.

### 2.2. Information Sources, Search Strategy, and Eligibility

PubMed/MEDLINE was searched for English-language literature concerning children and adolescents, principally 5–19 years, from 1 January 2015 through 29 August 2026. Earlier seminal sources were retained for definitions, life-course evidence, measurement, mechanisms, or foundational intervention findings. Search blocks combined bullying, peer victimization, cyberbullying, or cybervictimization with terms for age group, health outcomes, longitudinal evidence, intervention, healthcare, and population vulnerability. The full reproducible PubMed strings and search date appear in Supplementary Table S1. Google Scholar was used only for forward/backward citation tracing and locating known reports, not as a reproducible denominator, because its ranking and personalization are dynamic. Reference lists of major consensus reports and reviews [3,4,5,6,9,28,30,32,33] were examined. Named organizational sources were limited to WHO, UNESCO, CDC, the National Academies, and the American Psychological Association.

Eligibility and source selection. Eligible sources addressed traditional bullying, peer victimization, cyberbullying, or cybervictimization in children or adolescents and contributed directly to at least one review question. Eligible designs included systematic reviews, meta-analyses, longitudinal or nationally representative studies, intervention evaluations, measurement studies, and authoritative surveillance or clinical guidance. Sources focused exclusively on workplace bullying, university populations, or adults were excluded unless they provided direct life-course evidence; editorials without an evidentiary contribution and non-English reports were excluded. One author conducted the searches and preliminary relevance assessment; both authors reviewed the retained evidence set and resolved interpretive questions by discussion. Screening and extraction were not duplicated independently. The final synthesis comprised 61 sources: 53 peer-reviewed journal articles and eight institutional reports, guidance documents, or books. Because the original search was iterative and no prospective PRISMA-style database was maintained, unique records retrieved, deduplicated, and excluded cannot be reported defensibly. This is now stated explicitly and limits claims of completeness.

### 2.3. Data Charting, Narrative Synthesis, and Evidence Characterization

For each retained source, the authors charted the publication type, population, exposure definition, outcome domain, design, principal result, and limitation relevant to interpretation. The two authors determined the six synthesis domains through an iterative deductive–inductive process: the three review questions supplied the initial categories, and recurrent concepts identified during charting were compared, merged when redundant, or separated when they required distinct interpretation. Consensus produced six domains: conceptual boundaries and measurement; epidemiology; health and developmental consequences; mechanisms and modifiers; clinical identification and response; and multilevel prevention. No external experts, stakeholders, or formal consensus panel participated; the domains are therefore an author-developed organizational structure, not a validated taxonomy. This transparent, question-led synthesis is consistent with the purpose of a structured narrative review [31]. Numerical estimates were treated as context-dependent because the definitions, thresholds, recall periods, informants, platforms, cultures, and adjustment strategies differed. Source groups and their roles in the synthesis are summarized in Supplementary Table S2.

**Evidence characterization and SANRA self-assessment.** Inferential weight was characterized narratively rather than graded formally. Evidence was described as stronger when multiple meta-analyses or longitudinal studies converged; moderate when observational or review evidence was consistent but heterogeneous; and limited or emerging when evidence was cross-sectional, single-setting, indirect, or primarily conceptual. These labels are interpretive descriptors, not GRADE certainty ratings. Associations were not described as causal when bidirectionality or residual confounding remained plausible. The revised SANRA self-assessment is provided in Supplementary Table S3; the search item is conservatively scored 1 of 2 because the review did not use duplicate screening, formal risk-of-bias assessment, or a prospective screening log.

## 3. Narrative Synthesis Results

The synthesis identified six linked result domains. The findings are reported with quantitative estimates where comparable data were available; clinical interpretation and the author-developed framework are separated in Section 4, Section 5 and Section 6. Across the domains, confidence was highest for convergent associations supported by meta-analytic and longitudinal evidence, moderate for heterogeneous observational and intervention evidence, and limited for emerging digital harm and healthcare implementation strategies.

### 3.1. Conceptual Boundaries and Operational Definitions

**Bullying, power imbalance, and repetition.** The defining feature that separates bullying from ordinary peer conflict is the target’s reduced capacity to stop what is happening; repetition or a high likelihood of repetition and a power imbalance are core definitional elements [1,2]. A child facing a group, a socially dominant or physically stronger peer, or a person controlling humiliating images is not participating in an equal conflict [1,4,5]. Advice to ignore the behavior, retaliate, or enter face-to-face reconciliation without a safety assessment may reproduce the power imbalance; responses should instead be proportionate to risk and context [5,28,29].

Intent can be difficult to establish because aggressors may frame conduct as humor, banter, or retaliation while the targeted child experiences fear or humiliation [1,2,5]. Clinical care should therefore begin with the reported behavior, impact, power differential, and pattern rather than requiring proof of another person’s internal intention [5,28,29]. The term “bullying” should not be stretched to every reciprocal disagreement, but uncertainty about a research classification should not delay assessment of a harmful event [1,5,28].

**Peer victimization.** Peer victimization encompasses physical assault, verbal hostility, relational exclusion, rumor-spreading, property damage, sexual harassment, identity-based harassment, and digital aggression [1,5,9]. Some episodes satisfy the formal bullying definition; others may be one-time but severe [1,5]. In healthcare, the broader construct is clinically useful because urgent assessment is determined by harm and safety, not solely by whether repetition has already been documented [5,28,29].

**Cyberbullying within the peer ecology.** Cyberbullying shares substantial predictors and outcomes with traditional bullying [3,4,30]. Its distinctive affordances include potential anonymity, rapid dissemination, persistent and searchable content, large or uncertain audiences, around-the-clock access, and reduced control over copied material [4,30]. Networked redistribution can create repetition from one initial upload, while technical skill, access to private material, online popularity, or coordinated participation can create power asymmetry [1,4,30].

Online and offline peer aggression frequently overlap: school peers may continue aggression online, and digital content can reshape subsequent face-to-face relationships [3,4,30]. Conversely, an incident recorded at school may be redistributed and become a persistent digital exposure [4,30]. Clinical assessment should therefore ask about both contexts even when disclosure begins in only one [28,29,30].

**Role fluidity and clinical relevance.** Victim, perpetrator, bully–victim, and bystander or witness are analytical roles rather than fixed identities, and peer-group processes can sustain or interrupt bullying [5,34]. Person-first wording such as “a child who has bullied others” preserves accountability while avoiding a stable character label that may obscure co-occurring victimization, trauma, impulsivity, peer pressure, or family adversity [5,28,35].

Bully–victims—young people who are both targeted and aggressive toward others—show particularly adverse psychiatric and functional outcomes in longitudinal evidence [35]. Their aggression may follow different pathways; moral disengagement is one empirically examined mechanism in victim-to-perpetrator continuity [36]. Bystanders also occupy heterogeneous roles, including reinforcement, passive observation, defending, or reporting [34,37]. Assessment should therefore map current and previous roles, context, and transitions rather than assign one permanent label [5,28,34].

**Practical differential.** Table 1 summarizes the operational distinctions derived from surveillance definitions, foundational theory, and cyberbullying reviews [1,2,3,4,30].

### 3.2. Epidemiology and Measurement

Bullying prevalence depends on construct and threshold. UNESCO estimates that approximately one in three learners experiences bullying within a month across heterogeneous surveys [6]. In HBSC 2021–2022, 11% reported school bullying at least two or three times per month during the previous couple of months, while 15% reported cybervictimization at least once or twice during that period [7]. The United States Youth Risk Behavior Survey used a 12-month recall period and reported persistent school and electronic bullying [38]. Ariani et al. estimated pooled prevalences of 25% for victimization and 16% each for perpetration and bully–victim status [8]. These estimates answer different measurement questions and should not be pooled or compared as if thresholds, recall windows, age distributions, and sampling frames were equivalent.

Measurement choices can shift prevalence because instruments differ in whether they present a formal definition, list behaviors, require repetition or power imbalance, specify platforms, and distinguish victimization from perpetration [1,3,4,39,40,41]. Self-report can capture experiences hidden from adults but is affected by recall, interpretation, shame, and perceived anonymity; parent and teacher reports may miss covert relational or online conduct, while peer nominations raise confidentiality and feasibility issues [4,5,41]. Platform data identify observable content but cannot independently establish relational power or the target’s experience [4,30].

Sex and age patterns depend on the behavior, instrument, and setting [3,4,5,7]. Boys more often report physical aggression and some forms of perpetration, while girls may report more relational victimization or cybervictimization in some samples [5,7]. HBSC did not identify a uniform cross-national age gradient for bullying or cyberbullying involvement [7]; country-specific studies sometimes report declines in particular forms. Age and sex averages should therefore be attributed to their measures and never used as clinical stereotypes.

Digital measures require particular caution. Meta-analyses show wide prevalence ranges and substantial overlap between cyberbullying and traditional bullying [3,4]. Instruments developed around messages or social-network posts may not capture disappearing media, livestream harassment, location tracking, synthetic images, coordinated reporting, or gaming-based exclusion. The longitudinal meta-analysis cited in this review searched primary studies only through June 2021 [10]; it therefore informs prospective mental-health associations but not harm specific to newer generative or livestream technologies. Those emerging affordances are discussed as plausible, rapidly evolving exposures and are cross-referenced to the more recent conceptual review [30], not presented as established by the older evidence base.

For clinical practice, comprehensive validated questionnaires may support research or structured assessment, whereas brief confidential, behavior-specific questions may be more feasible for case-finding [28,29,39,40,42,43]. Questions should cover victimization and perpetration, online and offline contexts, impact, power, recurrence, and safety [1,28,29]. Because instruments differ substantially in constructs and psychometric support, selection should match the population and purpose, and a positive response should initiate clinical assessment rather than be treated as a diagnosis [39,40,41].

### 3.3. Integrated Health Pathways

Figure 1 is an author-developed conceptual synthesis guided by socioecological accounts of bullying [5,25,34,44], longitudinal evidence of bidirectionality [11], and evidence concerning psychological, digital, biological, sleep, educational, and life-course processes [9,12,17,18,19,30,45,46,47]. The exposure block refers specifically to physical, verbal, relational, and identity-based bullying or peer victimization, together with cyberaggression and exclusion. The middle block is labeled “candidate pathways” because mediation has not been tested for every proposed process within one causal model. Accordingly, the arrows represent evidence-informed, plausible associations and feedback loops, not proof that each pathway causally mediates every outcome.

**Threat appraisal and loss of control.** Repeated or persistent social threat may promote anticipatory vigilance, perceived uncontrollability, shame, self-blame, hopelessness, rumination, and negative beliefs [9,12,30,47]. These are candidate psychological pathways linking exposure with depression and anxiety; the present synthesis does not claim that each has been established as a statistical mediator [9,11,12].

Cyberbullying can intensify these appraisals because the audience is uncertain, evidence persists, and the home no longer guarantees respite. Yet, the digital context should not be treated as uniformly harmful. Digital spaces also provide friendship, identity exploration, peer support, and access to help. Harm depends on what happens online, to whom, under what conditions, and with what support, not simply on screen time [30].

**Behavioral pathways.** Avoidance, withdrawal, route changes, notification monitoring, substance use, and sleep disruption may reduce distress briefly while worsening attendance, participation, concentration, and connectedness [9,17,18,19]. These behaviors can plausibly reinforce isolation and functional impairment, although directionality may be reciprocal and varies across individuals [11,17,47].

**Social pathways.** Peer-group reinforcement, public humiliation, exclusion, and low confidence in adult help can alter reputation, belonging, and support [5,19,25,34]. Supportive peers, caregivers, and professionals may buffer harm through validation, connection, and coordinated safety planning [25,26,27,29,43], whereas disbelief or unsafe confrontation may deter disclosure or intensify distress [5,28,43].

**Biological embedding.** Chronic peer stress may become biologically embedded through dysregulation of stress-response, inflammatory, and sleep-related pathways. Longitudinal research has linked childhood victimization with higher later C-reactive protein levels, although patterns differ by bullying role, and confounding remains possible [45]. A systematic review of cortisol studies found that peer victimization was most consistently associated with blunted cortisol reactivity and a flatter diurnal slope, but methodological heterogeneity and inconsistent direction prevent a single biomarker interpretation [46]. These findings support biological plausibility, not a clinical laboratory test for bullying. No biomarker can confirm or exclude victimization, and the evidence should not be used to medicalize a fundamentally relational and structural exposure.

### 3.4. Health and Developmental Consequences

**Internalizing symptoms and mental disorders.** Bullying victimization is consistently associated with depression, anxiety, loneliness, reduced self-esteem, psychological distress, and post-traumatic stress symptoms [9,10,11,12]. A systematic review by Moore and colleagues concluded that victimization was associated with a broad range of adverse mental-health outcomes, while also emphasizing variation in study quality and the importance of longitudinal evidence [9]. An updated meta-analysis found bidirectional relationships between peer victimization and internalizing problems, suggesting both selection and social-causation processes [11].

Traditional and cybervictimization show overlapping but not necessarily identical associations with depression, suicidal ideation, suicide attempts, and self-harm [12]. A meta-analysis of 27 longitudinal studies involving 13,497 participants found a small, consistent prospective association between cybervictimization and later mental-health symptoms; its source search covered January 2010 through June 2021 [10]. The average magnitude is important at population level but does not imply that most exposed children develop a disorder. Chronicity, severity, baseline symptoms, support, and residual confounding remain important.

Self-esteem and life satisfaction are not secondary “soft” outcomes. Meta-analyses indicate that peer victimization is linked with lower self-esteem and internalizing symptoms, and that both victimization and perpetration are associated with lower life satisfaction [48,49]. These domains influence identity formation, help-seeking, social participation, and recovery [48,49].

**Self-harm and suicidality.** Involvement in bullying is associated with suicidal ideation and suicidal behavior across victims, perpetrators, and bully–victims [13]. The association is strongest and most concerning when victimization is frequent, when cyberbullying and traditional bullying co-occur, or when depression, hopelessness, sleep problems, family conflict, minority stress, or prior self-harm are present [12,13,14]. In a multi-country study of adolescents, the past-year prevalence of suicide attempts increased from 5.9% among those reporting no bullying to 32.7% among those bullied on 20–30 days in the previous month; the adjusted odds ratio was 5.51, indicating a dose-response pattern [14].

These associations must be communicated carefully: bullying does not make suicide inevitable, and suicidal behavior is multifactorial [13,14]. Deterministic language may stigmatize young people and obscure co-occurring risks. Nevertheless, bullying accompanied by hopelessness, self-harm, suicidal thoughts, severe withdrawal, escalating aggression, or an acute humiliating event warrants direct suicide-risk assessment and a proportionate safety response [13,14,28,29].

**Somatic symptoms, pain, and sleep.** Children often express distress through the body. Meta-analytic evidence links victimization with psychosomatic complaints such as headache, abdominal pain, dizziness, back pain, fatigue, poor appetite, and sleep difficulty [15]. Bullied children and adolescents also have a higher risk of recurrent headaches than non-bullied peers [16]. These symptoms are real and should not be dismissed as “just stress.” Clinical assessment must consider organic causes while also asking whether symptoms cluster around school days, transport, particular classes, extracurricular activities, or online events [15,16,28].

Sleep is both an outcome and a modifier. A recent meta-analysis supports bidirectional links between bullying involvement and sleep problems, with victimization predicting later sleep disturbance and sleep difficulty potentially increasing emotional dysregulation and vulnerability [17]. Sleep loss can worsen concentration, threat sensitivity, mood instability, and impulse control, thereby sustaining a harmful cycle [17]. Sleep assessment and treatment should therefore be incorporated into the care plan rather than treated as an incidental symptom [17,28,29].

**Externalizing behavior, substance use, and perpetration.** Bullying perpetration is associated with aggression, conduct problems, school disengagement, substance use, and later offending, although shared vulnerabilities and heterogeneity limit causal interpretation [9,35,50]. Some perpetrators are socially powerful, whereas others have experienced victimization, maltreatment, family conflict, or peer rejection [5,34,35]. Assessment should distinguish proactive from reactive aggression and examine group reinforcement, retaliation, and digital disinhibition [5,28,34].

Bully–victims merit particular attention because prospective evidence indicates broad adverse psychiatric outcomes among young people who were both victims and perpetrators [35]. Their profiles may include social vulnerability, emotion dysregulation, impulsivity, trauma exposure, or conflictual relationships [5,35,36]. A clinically defensible response therefore combines protection with accountability and individualized assessment [5,28,29].

**Education, relationships, and life-course health.** Bullying interferes with learning through fear, absence, reduced participation, concentration problems, and loss of school belonging [9,18,19]. Meta-analytic evidence links peer victimization with poorer academic achievement [18]. School effects can accumulate: missed instruction contributes to lower attainment; lower attainment narrows later opportunities; isolation limits social learning and support [18,19]. Bullying perpetration has also been associated prospectively with later offending, although shared risk factors require careful interpretation [50].

The consequences may extend well beyond adolescence. In a five-decade British birth cohort, frequent childhood victimization predicted elevated adult depression, anxiety, and suicidality and was associated with weaker social relationships, economic hardship, and poorer quality of life at age 50 [19]. Another prospective cohort found that chronic victimization predicted children being overweight at age 18 after accounting for earlier body size and multiple confounders [51]. These studies strengthen concern about long-term effects, but they should not be used to portray children as permanently damaged. Early recognition, supportive relationships, effective mental-health care, and safer environments can alter trajectories [5,25,28].

**Evidence map.** Table 2 maps the outcome domains to the strongest supporting evidence and makes the principal limitations explicit [9,10,11,12,13,14,15,16,17,18,19,35,45,46,48,49,50,51].

### 3.5. Vulnerability and Protection

Risk is not a property located solely within a child; socioecological evidence places bullying within interacting child, peer, family, school, digital, and societal conditions [5,20,21,22,23,24,25,26,27,34,44,47]. A visible or stigmatized difference becomes risk-amplifying in environments that reward aggression, exclude difference, or fail to protect the child [5,20,21,22,23,24,25].

**Neurodevelopmental differences, disability, and chronic illness.** Young people with autism, developmental delay, communication differences, attention difficulties, mobility limitations, visible conditions, or chronic illness may be targeted because they are perceived as different, have smaller peer networks, need assistance, or respond in ways peers exploit. A meta-analysis of school-aged youth with autism estimated pooled prevalences of 44% for victimization, 10% for perpetration, and 16% for bully–victim involvement [20]. In a nationally representative United States analysis, adolescents with chronic health conditions had higher victimization and perpetration than those without such conditions; young people with multiple conditions were 3.56 times as likely to experience victimization and 2.97 times as likely to perpetrate [21].

Healthcare teams may identify risk associated with chronic illness, disability, treatment burden, visible differences, assistive needs, or school absence because these factors are encountered during routine care [20,21,28,33]. Evidence-informed anticipatory guidance can ask how the condition is discussed at school, whether accommodations protect privacy and inclusion, and whom the child trusts; these are case-finding prompts rather than a validated universal screen [28,29,42].

**Identity, minority stress, and migration.** Identity-based bullying can compound ordinary peer aggression with minority stress and institutional discrimination. LGBTQ+ youth experience disproportionate cyberbullying and school victimization, with consequences shaped by family acceptance, school climate, and access to affirming support [22]. A meta-analysis of minority stressors found that bias-based victimization, general victimization, bullying, and negative family treatment were associated with suicidal ideation and attempts among sexual and gender minority youth [23]. The appropriate clinical response is not to pathologize identity but to address the hostile environment, strengthen affirming relationships, and assess whether disclosure to caregivers is safe.

Evidence regarding migrant and refugee youth is heterogeneous. First-generation immigrant status does not create uniform risk; language, racialization, legal insecurity, socioeconomic conditions, trauma history, school composition, and inclusion practices modify exposure [24]. Clinicians should ask about racism, accent-based harassment, religion, clothing, cultural exclusion, and threats related to immigration or conflict without assuming that all minority youth share the same experience [5,24,28].

**Appearance, body size, and social status.** Appearance, body size, visible conditions, pubertal development, clothing, and socioeconomic markers can become targets within stigmatizing peer environments [5,9,28]. Associated shame may affect participation, body image, treatment adherence, and care-seeking [9,28]. Clinicians should reject stigma and address independent health needs without implying that changing the child is the solution to peer aggression [5,28].

**Protective relationships and environments.** Protective factors operate across levels. Meta-analytic synthesis identifies empathy, prosocial peers, social competence, supportive parenting, positive school climate, and school connectedness as recurrent buffers against bullying and cyberbullying [25]. Parent–child attachment and communication may reduce the mental-health impact of cybervictimization [26,27]. Protection is not equivalent to teaching the child to tolerate abuse. It means creating credible routes to safety, belonging, and help.

Table 3 synthesizes risk-amplifying and protective conditions from systematic, meta-analytic, and observational evidence [5,11,20,21,22,23,24,25,26,27,34,37,44,47,52]. Citations are provided within each row; the clinical implications are author-derived applications of that evidence rather than validated decision rules.

### 3.6. Cyberbullying in a Changing Digital Environment

**Distinctive digital affordances.** Cyberbullying may involve an uncertain audience, reproducible and searchable content, persistence across platforms, and reduced control over redistribution; these affordances can sustain vigilance and humiliation [4,30]. Removal from one platform does not guarantee that copies have disappeared [4,30].

Anonymity can reduce social cues and accountability, although aggressors are often known peers. Group chats, public comments, exclusion, impersonation, location tools, and gaming environments can amplify harm [30]. Synthetic images, fabricated messages, and realistic audio or video are emerging risks [30]. Because direct outcome evidence for these generative affordances remains limited and postdates the search window of the cited longitudinal meta-analysis [10], they are treated here as emerging concerns requiring surveillance and validation rather than quantified causal claims [10,30].

**Risks of blanket device removal.** Automatic device confiscation may remove social support or school access and discourage disclosure; youth perspectives and digital-health guidance favor collaborative, proportionate responses [30,43,47,55,56]. When immediate danger does not require restriction, reasonable actions include preserving appropriate evidence, securing accounts, blocking or muting aggressors, reporting harmful content, involving the school when relevant, and agreeing with the child on feasible changes [5,28,29,30,56].

**Co-occurring digital risks.** Cyberbullying may overlap with sexual-image abuse, grooming, stalking, extortion, impersonation, hate speech, or threats of physical harm [5,28,30]. These exposures may trigger specific safeguarding or legal duties, depending on jurisdiction [5,28,29]. Assessment should clarify sexual content, adult involvement, location exposure, compromised accounts, and threat credibility; harmful content should not be repeatedly viewed or redistributed [5,28,29,56].

**Avoiding digital moral panic.** The association between general digital-technology use and adolescent well-being is small and heterogeneous, and person-specific vulnerability matters [30,47]. Cyberbullying is a harmful experience but it should not be conflated with all social-media use. Social platforms can sustain friendships, connect marginalized youth with affirming communities, and facilitate help-seeking. Clinical advice should focus on experiences, content, displacement of sleep or activity, compulsive patterns, and loss of control rather than a single universal screen-time threshold [47,55,56].

## 4. Discussion and Clinical Implications

### 4.1. Targeted Case-Finding and Clinical Inquiry

Bullying and broader peer harm should enter the differential when a child presents with recurrent unexplained somatic complaints, sleep disturbance, school refusal or absence, declining performance, unexplained injuries, social withdrawal, marked distress after device use, disordered eating, anxiety, depression, aggression, substance use, self-harm, or suicidality [9,12,13,14,15,16,17,18,19,28,35,50]. Behavior-specific inquiry may also be reasonable for children with relevant contextual vulnerability or recent transitions [20,21,22,23,24,25,26,27,28,29,42]. This is targeted case-finding, not a recommendation for universal screening.

Pediatric primary-care providers report variable screening practices, and stronger professional attitudes and self-efficacy—not knowledge alone—have been associated with a greater likelihood of asking [42]. Youth themselves have indicated that they value healthcare professionals asking about bullying and may not realize the issue is within the clinician’s role [43]. This creates a simple but important opportunity: permission to disclose often begins when a trusted adult asks calmly and specifically.

**How to ask.** Developmentally appropriate private discussion, a clear explanation of confidentiality and its limits, and behavior-specific wording can facilitate disclosure [28,29,42,43]. Asking only, “Are you being bullied?” may miss children who do not use or accept that label [29,43]. The following prompts are examples derived from pediatric and youth-perspective literature, not a validated screening instrument [28,29,42,43]:

- “Has anyone at school, in your neighborhood, in a team, or online repeatedly embarrassed, threatened, excluded, hit, or pressured you?”
- “Is there anyone you try to avoid because you feel unsafe, humiliated, or powerless around them?”
- “Has anyone shared or threatened to share messages, images, videos, or personal information about you?”
- “Have you joined in when someone else was targeted, or have you hurt someone online or in person?”
- “Who knows about this? Who has helped? What happened after you told an adult?”
- “What are you most worried will happen next?”

The tone and follow-through influence whether disclosure feels safe [29,43]. A validating response can acknowledge the report, reduce blame, and invite collaborative safety planning. Clinicians should not promise complete secrecy, immediate resolution, or a predetermined institutional outcome [28,29,43].

### 4.2. Exposure Characterization and Immediate Safety

Assessment should characterize behavior, onset, frequency, duration, escalation, locations and platforms, power imbalance, relationship to the aggressor, audience, bystander roles, content persistence, prior help-seeking, and institutional responses [1,5,28,29]. Victimization, perpetration, and witnessing should be asked about separately because roles can overlap [34,35]. Identity-based, sexual, dating-related, extortive, or adult-involved behavior requires differentiated safeguarding assessment [5,22,23,28,29].

**Health, function, and immediate safety.** Assessment should cover injury, pain, mood, anxiety, trauma symptoms, sleep, appetite, attendance, concentration, academic decline, withdrawal, aggression, substance use, self-harm, suicidal ideation, and access to means [9,12,13,14,15,16,17,18,28,29]. Active suicidal intent, severe injury, an unsafe destination, sexual-image abuse, extortion, credible threats, weapons, or adult involvement warrants urgent action under local emergency and safeguarding procedures [5,13,14,28,29].

Validated measures can support but cannot replace clinical assessment. The Revised Olweus Bully/Victim Questionnaire assesses traditional bullying roles [39], while the European Cyberbullying Intervention Project Questionnaire assesses cybervictimization and cyberaggression [40]. Other available measures include the Cyberbullying and Online Aggression Survey and multiple behavior- or technology-specific questionnaires; a systematic review identified 44 instruments and substantial variation in definitions and psychometric reporting [41]. Thus, the ECIP-Q is one example rather than a uniquely preferred instrument. Selection should be purpose-, age-, language-, and culture-specific, and no questionnaire by itself establishes causation or a safeguarding threshold [39,40,41].

### 4.3. Documentation and Clinical Actions

Documentation should use the child’s own words when possible, describe observable injuries and symptoms, record dates and platforms, distinguish reported information from verified findings, document suicide and safety assessment, and state the agreed plan [5,28,29]. Digital evidence should be handled under institutional privacy and safeguarding procedures; sexual images of minors should not be copied into an ordinary clinical record [5,28,29].

Table 4 translates pediatric, outcome, and measurement evidence into potential assessment prompts [13,14,15,16,17,18,19,28,29,35,39,40,42,43,50,54]. Citations are provided within each row; the prompts and responses remain author-derived and have not been validated as a screening instrument or clinical decision rule.

## 5. Proposed BV-CARE Conceptual Framework

BV-CARE is an author-developed conceptual framework, not a validated screening instrument, decision rule, guideline, or standard of care. It is intended to organize clinical reasoning after bullying is suspected or disclosed. No major guideline body identified in this review recommends universal bullying screening; accordingly, BV-CARE should not be interpreted as such a recommendation.

### 5.1. Framework Development and Evidentiary Status

The two authors drafted the framework by mapping recurrent clinical tasks across three evidence streams: recognition and youth disclosure [28,42,43], safety assessment and symptom management [9,10,11,12,13,14,15,16,17,28,29,39,40,54], and coordinated intervention and follow-up [5,29,32,33,37,42,44,52,53,54,57,58]. They iteratively grouped overlapping tasks, ordered them from inquiry to reassessment, and selected a mnemonic for recall. No Delphi process, external expert panel, patient or youth co-design, formal content-validity study, or empirical testing was undertaken. The source-to-step mapping in Table 5 therefore documents conceptual provenance, not validation. The recommendations in this section are explicitly presented as author-derived propositions where effectiveness or feasibility evidence is absent.

Table 5 maps each BV-CARE step to the evidence domains that informed it. The sequence and mnemonic are author-developed and non-validated [5,9,10,11,12,13,14,15,16,17,28,29,32,33,37,39,40,42,43,44,52,53,54,57,58].

### 5.2. Collaborative Planning and Coordinated Support

Children may avoid disclosure because they anticipate retaliation, device loss, parental overreaction, public confrontation, or disruptive school changes [29,43]. Collaborative planning should ask what outcome the child wants, whom they trust, what they fear will happen, and which actions feel safe [28,29,43]. Participation does not transfer adult responsibility to the child; it uses the child’s contextual knowledge while adults retain safeguarding duties [5,28,29].

**Family involvement.** Caregiver support can buffer the mental-health impact of victimization, but disclosure should be planned cautiously if it could lead to rejection, violence, punishment, or unsafe disclosure of sexual orientation or gender identity [22,23,26,27]. When involvement is safe, calm listening, validation, documentation, and coordinated school contact align with family- and child-centered recommendations [5,28,29,43,52]. Hostile contact or public posting may escalate exposure and should be avoided [5,28,29].

**School and interprofessional coordination.** Clinicians do not determine school disciplinary guilt; their role is to communicate health and safety needs, with appropriate consent, and support a proportionate plan [5,28,29]. Relevant measures may include a designated school contact, safer transitions and supervised spaces, temporary academic adjustments, symptom and attendance monitoring, mental-health treatment, and safeguarding or social-work coordination [5,28,29,33,54,60].

**Supporting children who have bullied others.** Evidence supports combining accountability with assessment of aggression type, co-occurring victimization, peer reinforcement, family adversity, substance use, and mental-health needs [5,28,34,35,36,50]. Consequences should be proportionate and linked to behavior change; repair should occur only when safe and voluntary. Forced apology or face-to-face mediation is unsuitable when coercion or a power imbalance persists because it may reproduce the unsafe dynamic [5,28,29].

### 5.3. Validation Roadmap

Because BV-CARE is conceptual, validation should proceed in stages consistent with instrument-development and implementation-science guidance: multidisciplinary and youth/caregiver content review; cognitive interviews for comprehensibility and acceptability; feasibility testing across diverse clinical settings; and prospective pilot evaluation with pre-specified clinical, implementation, equity, and adverse-event outcomes [29,43,54,59,61]. Reliability or diagnostic-accuracy testing should be considered only after the construct and intended use are fixed [61]. Comparative implementation studies should evaluate fidelity, acceptability, feasibility, referral completion, safety planning, symptom and attendance recovery, recurrence, retaliation, and unintended harm before routine or universal use is proposed [29,54,59].

## 6. Intervention Evidence and System-Level Implications

### 6.1. Multilevel Intervention Effectiveness

School-based anti-bullying programs work on average, but the effects are modest and heterogeneous. A large Campbell systematic review found average reductions of approximately 18–19% in perpetration and 15–16% in victimization [32]. Another meta-analysis reported benefits for bullying, cyberbullying, and mental-health outcomes, while underscoring variation in program content and study quality [53]. For cyberbullying, Polanin and colleagues found small but significant reductions in perpetration (Hedges’ g = −0.18) and victimization (g = −0.13) [57]. Educational interventions likewise yield very small to small effects across traditional and cyberbullying outcomes [58]. Programs grounded in socioecological principles appear stronger when they include interpersonal interaction and meaningful stakeholder agency rather than information delivery alone [44]. Broader adolescent mental-health guidance likewise favors developmentally appropriate promotive and preventive interventions embedded in families, schools, communities, and services [60].

Modest average effects are compatible with a complex, multilevel phenomenon maintained by peer-group, school, family, social, and digital conditions [5,32,34,44,53,57,58]. The intervention evidence therefore favors sustained, layered approaches and implementation monitoring rather than expecting a brief educational component to eliminate bullying [5,32,44,53,57,58].

**Whole-school approaches.** Multicomponent programs commonly combine leadership, consistent definitions and reporting, staff training, supervision, student participation, parent engagement, social-emotional learning, and monitoring [5,32,44,53]. Peer processes require attention because reinforcement can sustain aggression and defending can interrupt it [34]; school programs can also improve cyber-bystander behavior, although effects vary [37].

Policy alone is unlikely to change behavior if reporting is inaccessible or students anticipate disbelief or retaliation [5,32,44]. Reporting routes should be confidential, accessible, and connected to visible action. Restorative processes should be considered only when safety is established, responsibility is acknowledged, participation is voluntary, and the power imbalance will not be reproduced [5,28,29].

**Family-focused prevention.** Parenting programs can reduce bullying involvement, particularly when they strengthen warmth, monitoring, communication, non-violent discipline, and problem-solving [52]. Developmentally appropriate digital guidance should combine privacy and security with a clear route to help that does not automatically remove supportive online access [30,47,55,56].

### 6.2. Healthcare and Nursing Interventions

For this paper, healthcare contributions are organized as universal anticipatory guidance, selective case-finding for groups with elevated exposure or vulnerability, and indicated intervention after disclosure or clinical presentation [28,29,33,60]. These tiers are an author-applied prevention structure; their specific bullying components require implementation evaluation rather than being interpreted as a validated clinical standard [29,33,54].

A systematic review of nursing interventions identified contributions in awareness, education, coping and social skills, screening, support, and coordination [33]. Nurses may be among the first professionals to connect recurrent symptoms with peer harm because they encounter children in schools, primary care, emergency services, inpatient units, community programs, and mental-health settings. Their role is strongest when embedded in an interprofessional pathway rather than limited to a one-time educational session.

Quality-improvement evidence shows that bullying inquiry can be integrated into pediatric specialty care when staff training, prompts, triage, and referral pathways are available [54]. Identification without response capacity may create implementation and trust risks; services should predefine responsibility for positive findings, urgent-risk escalation, referral, school communication, and follow-up [29,54,59]. The evidence supports testing complete pathways, not treating screening completion as the clinical endpoint [29,54].

### 6.3. Tiered Action Matrix

Table 6 distinguishes evidence-supported intervention levels from proposed healthcare contributions and candidate evaluation indicators [28,29,32,33,37,42,43,44,52,53,54,57,58].

## 7. Prioritized Research and Policy Agenda

The priorities in this paper are author-derived from four explicit criteria: the magnitude or severity of the documented health burden; uncertainty that currently limits causal or clinical interpretation; the potential for findings to change prevention or healthcare practice; and the need to evaluate equity and unintended harm [5,9,10,11,20,21,22,23,24,28,29,30,31,32,33,42,54]. Applying these criteria yielded three connected areas: stronger trajectory and measurement research; rigorous evaluation of healthcare pathways; and equitable youth co-design with platform accountability. This ordering is a transparent interpretation of the reviewed evidence, not a formal consensus ranking or externally validated priority-setting exercise.

### 7.1. Trajectories, Measurement, and Causal Inference

Cross-sectional studies dominate important parts of the literature, while fewer studies resolve onset, persistence, desistance, role transition, recovery, and reciprocal effects [3,4,9,10,11]. Prospective research should measure traditional and digital experiences together, establish baseline mental health, model within-person change and bidirectionality, and identify modifiable responses associated with recovery [10,11,17,19]. This priority follows directly from the causal uncertainty identified across outcome domains.

**Measurement standardization.** Comparability requires explicit reporting of definitions, behavior items, power and repetition criteria, recall period, informant, platform, and role classification [1,3,4,41]. A common core could improve comparison, while optional modules capture emerging forms such as synthetic-media humiliation, impersonation, coordinated harassment, doxxing, location-based threats, and immersive-environment aggression [30,41]. Measures require developmental, linguistic, cultural, and disability-sensitive validation before cross-group interpretation [39,40,41,61].

**Cross-cutting design considerations.** Prospective cohorts, repeated within-person measurements, quasi-experimental policy evaluations, appropriate negative controls, and adjustment for baseline mental health and family adversity would strengthen causal inference [10,11,19]. Researchers should pre-specify constructs and avoid causal language for cross-sectional associations [11,31]. New digital modules should undergo transparent content and psychometric validation rather than being assumed equivalent to established measures [30,41,61].

### 7.2. Healthcare Pathway Evaluation

Healthcare pathway evaluation is prioritized because provider practice is variable, youth welcome appropriate inquiry, and current studies establish feasibility more clearly than patient benefit or safety [42,43,54]. Trials and implementation studies should test behavior-specific case-finding, risk stratification, documentation, nurse-led coordination, referral, and follow-up as complete pathways [29,33,54,59]. Outcomes should include safety planning, referral completion, symptoms, attendance, recurrence, retaliation, equity, family experience, fidelity, capacity constraints, and unintended harm [29,54,59].

### 7.3. Equity, Youth Co-Design, and Platform Accountability

Equity is prioritized because exposure and consequences differ across disability, chronic illness, sexual and gender minority status, migration context, socioeconomic conditions, and school climate [20,21,22,23,24,25,26,27]. Youth and caregiver involvement should be used to test whether inquiry, confidentiality, digital-safety actions, and outcome measures are acceptable and safe [43,59,61]. Co-design should therefore be embedded from content development through implementation evaluation rather than added after a pathway is fixed.

**Platform accountability.** Platform features can shape persistence, audience reach, privacy, amplification, and access to reporting [4,30,47,55,56]. Evaluation should examine reporting usability, response time, content persistence, recommender amplification, privacy defaults, age-appropriate design, and victim support. Automated moderation may introduce false positives, context loss, cultural bias, and surveillance concerns; these risks justify transparent appeal, human review, and child-safety expertise [30,47,56]. Platform action complements—rather than replaces—family, school, healthcare, and public-policy responsibilities [5,30,56].

## 8. Strengths and Limitations

The principal strength of this paper is its explicitly structured integration of related but non-identical exposures across mental, physical, developmental, and life-course outcomes. Quantitative estimates are linked to measurement limits, evidentiary weight is interpreted by design and consistency, and evidence-supported findings are separated from author-derived clinical propositions. The revised manuscript also reports full search strings, source composition, source-to-table traceability, and a conservative SANRA self-assessment [31].

The limitations materially constrain interpretation. Only PubMed/MEDLINE was used as a reproducible bibliographic database; Google Scholar served citation tracing and known-item retrieval, so nursing, education, and psychological literature not indexed in PubMed may have been missed. The search was English-only, iterative, and not prospectively logged; unique retrieval and exclusion counts cannot be reconstructed, and neither screening nor data charting was independently duplicated. No protocol was registered, no formal risk-of-bias tool was applied to each source, and no certainty-of-evidence framework or new meta-analysis was undertaken. Narrative evidence labels may reflect author judgment, and source selection is vulnerable to availability and citation bias. Heterogeneous definitions, thresholds, recall periods, informants, cultures, and digital environments limit comparability. Most health associations are observational and remain susceptible to reverse causation and residual confounding; intervention averages conceal variable fidelity and cultural fit. Evidence for healthcare screening effectiveness, feasibility, and harm is sparse, and no identified major guideline recommends universal screening. BV-CARE was developed by the authors without external consensus, youth co-design, or empirical testing and must be regarded as hypothesis-generating. Legal and safeguarding duties vary by jurisdiction. These constraints reduce generalizability and require cautious, context-specific application.

## 9. Conclusions

Bullying, peer victimization, and cyberbullying are interconnected but operationally distinct exposures. Convergent evidence supports associations with internalizing symptoms, suicidality, psychosomatic complaints, sleep disturbance, and impaired functioning, while longitudinal and intervention findings remain modest and heterogeneous. Measurement differences, bidirectionality, and residual confounding preclude simple causal or prevalence comparisons.

The defensible clinical implication is targeted case-finding: when symptoms, functional change, injury, school avoidance, digital distress, or contextual risk raise concern, clinicians should consider private, behavior-specific inquiry and assess immediate safety, self-harm, suicide risk, physical and mental-health effects, and functioning. Evidence does not yet justify universal bullying screening. Any inquiry should be linked to a response pathway and adequate referral capacity.

BV-CARE offers a hypothesis-generating structure for organizing such a response, but it is not ready for routine implementation as a validated clinical model. Its value, acceptability, equity, feasibility, and unintended effects require stakeholder co-design and prospective evaluation. Until then, clinicians should apply established local safeguarding, suicide-risk, mental-health, and child-protection procedures and use the framework only as a conceptual aid.

## Figures and Tables

**Figure 1:**
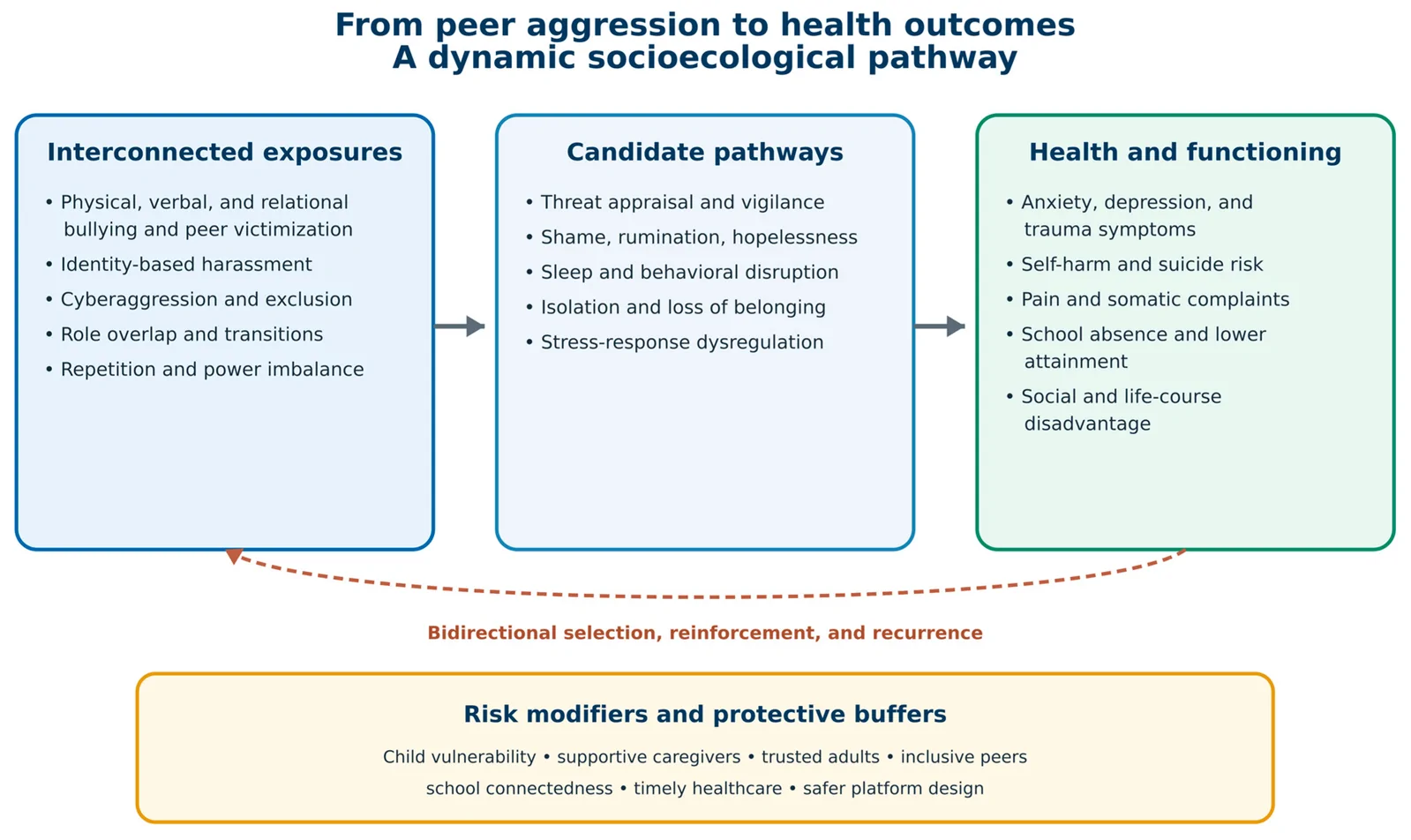
Evidence-informed, author-developed socioecological pathway linking specified forms of peer aggression with candidate psychological, behavioral, social, educational, and biological processes and health or functioning outcomes. Bidirectionality and multilevel modifiers are supported conceptually and empirically, but the complete model has not undergone formal mediation testing [5,9,11,17,18,19,25,26,27,30,34,44,45,46,47].

**Table 1:** Conceptual distinctions relevant to research classification and clinical response.

Construct	Core Features	Typical Examples	Clinical Implication
Bullying	Unwanted aggression; power imbalance; repeated or likely to recur	Repeated threats, exclusion, humiliation, physical intimidation	Assess pattern, power, safety, health effects, and system response
Peer victimization	Harm by peers; may or may not meet repetition/power criteria	One severe assault, rumor, identity-based slur, social exclusion	Do not withhold care because a formal bullying threshold is uncertain
Cyberbullying	Digitally mediated aggression with potential persistence and networked repetition	Harassing messages, impersonation, non-consensual image sharing, coordinated exclusion	Ask about audience, anonymity, saved evidence, account security, and content persistence
Peer conflict	Disagreement between peers of broadly similar power	Argument, reciprocal dispute, friendship rupture	Support problem solving; reassess if coercion, fear, or imbalance emerges
Dating or sexual violence	Aggression within dating/sexual context; may involve coercion	Threats by a dating partner, sexual-image coercion	Follow specific safeguarding and violence protocols; do not subsume under bullying
Hate- or identity-based harassment	Harm targeting protected or stigmatized identity	Racist, ableist, homophobic, transphobic, religious harassment	Assess cumulative discrimination, minority stress, family/community safety, and institutional duties

**Table 2:** Evidence map of major health and developmental outcomes associated with bullying involvement.

Outcome Domain	Consistent Findings	Important Cautions	Clinical Relevance
Depression and anxiety	Cross-sectional, longitudinal, and meta-analytic associations; bidirectionality supported [9,10,11,12]	Baseline symptoms and shared vulnerabilities partly explain associations	Assess current symptoms, function, duration, and prior history
Self-harm and suicidality	Increased risk across roles; dose-response with frequent victimization [13,14]	Suicide is multifactorial; avoid single-cause attribution	Ask directly and urgently when warning signs are present
Somatic symptoms and pain	Higher psychosomatic complaints and headache risk [15,16]	Continue appropriate medical differential diagnosis	Ask about timing, school pattern, sleep, fear, and online stress
Sleep	Bidirectional association with victimization/perpetration [17]	Device use, depression, family stress, and school schedule may confound	Include sleep hygiene and treatment in the plan
Externalizing behavior	Aggression, conduct problems, and later offending are associated with perpetration [35,50]	Perpetrators are heterogeneous; not all have psychopathology	Combine accountability, behavioral assessment, and support
Academic and social functioning	Absence, lower achievement, reduced belonging, and isolation [18,19]	School climate and pre-existing learning needs matter	Coordinate attendance, accommodations, safety, and reintegration
Adult health and socioeconomic outcomes	Persistent mental-health, relationship, quality-of-life, and economic associations [19,35,51]	Observational cohorts cannot eliminate all confounding	Early intervention may prevent cumulative disadvantage
Biological markers	Cortisol and inflammatory differences reported [45,46]	Heterogeneous, non-specific, not diagnostically useful	Supports stress plausibility; no biomarker testing is indicated

**Table 3:** Socioecological risk-amplifying and protective conditions.

Level	Risk-Amplifying Conditions	Protective Conditions	Implication for Care
Child	Prior anxiety/depression, impulsivity, communication difficulty, visible difference, limited coping repertoire [11,20,21,35,48]	Emotion regulation, self-compassion, problem-solving, help-seeking confidence [25]	Treat symptoms and build skills without blaming the child [5,28,29]
Peer	Aggressive norms, popularity rewards, group reinforcement, isolation [25,34]	Defending peers, inclusive friendships, prosocial norms [25,34,37]	Ask who witnessed, reinforced, defended, or can provide support [28,29,43]
Family	Conflict, harsh parenting, low monitoring, unsafe identity disclosure [26,27,52]	Warm communication, predictable support, developmentally appropriate digital involvement [25,26,27,52]	Engage caregivers when safe; coach calm, validating responses [26,27,29,52]
School	Poor supervision, inconsistent reporting, discriminatory climate, punitive-only response [5,6,32,44,53]	Connectedness, clear policy, trained staff, confidential reporting, restorative and protective practices [5,25,32,44,53]	Coordinate a documented safety and education plan [28,29,33,54]
Digital	Anonymity, amplification, persistent content, weak privacy, coordinated harassment [4,30,47]	Privacy controls, evidence preservation, platform reporting, digital literacy, supportive online communities [30,47,55,56]	Address security and content while preserving healthy connection [29,30,56]
Society	Stigma, inequality, discriminatory policy, fragmented services [5,6,22,23,24]	Child-rights protections, inclusive policy, accessible mental-health care, cross-sector accountability [5,6,25]	Advocate beyond the individual case and monitor inequities [5,28,33]

**Table 4:** Clinical presentations, assessment prompts, and immediate actions.

Presentation or Clue	Questions to Consider	Potential Clinical Response
Headache, abdominal pain, fatigue, dizziness [15,16]	Do symptoms cluster before school, transport, sport, or particular classes? [15,16,28]	Assess medical causes and psychosocial context in parallel [28,29]
School refusal, absence, falling grades [18,19]	Is the child avoiding a person, place, route, changing room, or online spillover? [18,19,28]	Coordinate safety, attendance, and educational support [5,28,29]
Sleep disturbance or nighttime device checking [17]	Is the child monitoring threats, fearing new posts, or ruminating? [17,30,47]	Assess mental health and digital exposure; treat sleep [17,28,29]
Withdrawal or loss of friends [9,19,48]	Was the child excluded, publicly humiliated, impersonated, or pressured? [4,9,30]	Map peer network and identify a trusted ally [25,28,29,43]
Irritability, aggression, disciplinary incidents [35,50]	Is the child also being targeted? Is aggression reactive or proactive? [34,35,50]	Assess both victimization and perpetration; protect and hold accountable [5,28,29]
Distress after online activity [4,10,30]	What platform, content, audience, persistence, and anonymity are involved? [4,30]	Preserve evidence safely, secure accounts, report content, assess escalation [28,29,30,56]
Self-harm, hopelessness, suicidal thoughts [13,14]	Are there plans, intent, means, prior attempts, acute humiliation, or perceived inescapability? [13,14,29]	Immediate suicide-risk assessment and safety intervention [13,14,28,29]
Sexual image, extortion, stalking, adult contact [5,28,30]	Was content coerced or non-consensual? Is location known or a threat credible? [5,28,30]	Follow urgent safeguarding and jurisdiction-specific procedures [5,28,29]

**Table 5:** The proposed BV-CARE clinical response framework.

Step	Proposed Actions and Evidence Basis	Actions to Avoid or Evaluate Cautiously
**B—Bring up bullying confidentially**	Ask routinely when clinically indicated; use behavior-based online/offline questions; include victimization, perpetration, and witnessing; explain confidentiality [28,29,42,43]	Waiting for the child to use the word “bullying”; asking only in front of caregivers [28,29,43]
**V—Validate and characterize**	Believe the disclosure; reduce blame; clarify pattern, power, severity, roles, platforms, audience, identity-based content, and prior responses [5,28,29,43]	Minimizing as “banter”; blaming the child’s appearance, identity, disability, or device use [5,28,29,43]
**C—Check safety and health effects**	Assess injuries, threats, sexual or extortive content, adult involvement, mood, trauma symptoms, sleep, substance use, self-harm, suicide risk, and functioning [9,12,13,14,15,16,17,28,29]	Assuming bullying alone explains every symptom; omitting direct suicide questions [13,14,28,29]
**A—Activate coordinated supports**	Agree on immediate safety; identify a trusted adult; involve caregivers when safe; communicate with school/safeguarding teams; address digital security and evidence [5,28,29,33,54,56]	Forced confrontation or mediation where power imbalance persists; indiscriminate device confiscation [5,28,29,56]
**R—Refer and treat**	Treat physical and mental-health consequences; refer to psychology, psychiatry, social work, school nursing, safeguarding, or specialist services as indicated [28,29,33,54]	Referring externally without an interim plan or warm handoff [29,33,54]
**E—Evaluate recovery and recurrence**	Schedule follow-up; reassess exposure, symptoms, attendance, sleep, treatment, school response, retaliation, and new platforms; escalate if unresolved [29,33,54,59]	Treating one conversation or one report as closure [29,54,59]

Note: Bold type identifies the six mnemonic step labels of BV-CARE.

**Table 6:** Tiered prevention and response matrix.

Prevention Level	Target Population	Evidence-Supported or Proposed Actions	Potential Healthcare Contribution	Candidate Evaluation Indicators
Universal	All children, families, and school communities	Safe climate, clear policy, digital citizenship, social–emotional learning, bystander skills, confidential reporting	Anticipatory guidance; staff education; public-health advocacy	Greater help-seeking and connectedness; reduced bullying exposure
Selective	Groups with elevated exposure or vulnerability	Inclusive accommodations, mentoring, caregiver coaching, transition planning, targeted peer support	Proactive inquiry; condition-specific counseling; liaison with school	Earlier identification; fewer recurrent symptoms and absences
Indicated	Identified victim, perpetrator, bully–victim, or distressed witness	Individual safety plan, family-school coordination, behavioral intervention, content removal/security, mental-health care	BV-CARE assessment; treatment; referral; documentation; follow-up	Safety restored; symptoms and recurrence reduced; functioning improves
Crisis	Immediate danger, suicidality, severe injury, sexual-image abuse, extortion, credible threat, adult involvement	Emergency protection, suicide intervention, safeguarding, jurisdiction-specific reporting	Stabilize; protect; preserve appropriate evidence; warm handoff	Immediate risk contained; continuity of care confirmed

## Data Availability

No new data were created or analyzed in this study. Data sharing is not applicable to this article.

## Supplementary Materials

The following supporting information can be downloaded at: https://www.mdpi.com/article/10.3390/healthcare14193180/s1, Table S1. Reproducible search strategy and source-specific use. Table S2. Evidence-source groups and role in the synthesis. Table S3. SANRA self-assessment after revision.
