# Supplementary material for: Bullying and Cyberbullying in Childhood and Adolescence: A Narrative Review and Proposed Clinical Response Framework
Source: Healthcare (Basel). 2026 Sep 25;14(19):3180. doi: 10.3390/healthcare14193180 (PMC13665281; doi:10.3390/healthcare14193180)
Supplement: Supplementary file 1 [file healthcare-14-03180-s001.zip › healthcare-4563227-supplementary.pdf]

Supplementary Materials

Table S1. Reproducible search strategy and source-specific use.

Source	Date	Search strategy	Role and denominator
PubMed/MEDLINE	29 August 2026	(bullying[Title/Abstract] OR "peer victimization"[Title/Abstract] OR cyberbullying[Title/Abstract] OR cybervictimization[Title/Abstract]) AND (child*[Title/Abstract] OR adolescen*[Title/Abstract] OR youth[Title/Abstract]) AND (health[Title/Abstract] OR mental[Title/Abstract] OR somatic[Title/Abstract] OR sleep[Title/Abstract] OR suicid*[Title/Abstract] OR self-harm[Title/Abstract])	Primary reproducible database search; no defensible record denominator because the original iterative search was not prospectively logged.
PubMed/MEDLINE	29 August 2026	(bullying[Title/Abstract] OR cyberbullying[Title/Abstract]) AND (child*[Title/Abstract] OR adolescen*[Title/Abstract]) AND (longitudinal[Title/Abstract] OR meta-analysis[Publication Type] OR systematic review[Publication Type])	Higher-inference longitudinal and review evidence.
PubMed/MEDLINE	29 August 2026	(bullying[Title/Abstract] OR cyberbullying[Title/Abstract]) AND (intervention*[Title/Abstract] OR prevention[Title/Abstract] OR screening[Title/Abstract]) AND (school*[Title/Abstract] OR pediatric*[Title/Abstract] OR healthcare[Title/Abstract] OR nurse*[Title/Abstract])	Intervention and healthcare translation evidence.
PubMed/MEDLINE	29 August 2026	(bullying[Title/Abstract] OR cyberbullying[Title/Abstract]) AND (autism[Title/Abstract] OR disabilit*[Title/Abstract] OR "chronic illness"[Title/Abstract] OR LGBTQ[Title/Abstract] OR migrant*[Title/Abstract])	Vulnerability and equity evidence.
Google Scholar	Updated through 29 August 2026	Forward and backward citation tracing from included reviews and retrieval of known titles or organizational reports.	Supplementary only; dynamic ranking was not treated as a reproducible denominator.
Organizational websites	Updated through	Named-item searches of WHO, UNESCO, CDC, National Academies, and American Psychological Association resources.	Surveillance, definitions, policy, and

Source	Date	Search strategy	Role and denominator
	29 August 2026		guidance; eight non-journal sources were retained in the 58-source set.

Note: The search description reports only procedures that can be supported by the retained manuscript record; unlogged record counts were not reconstructed.

Table S2. Evidence-source groups and role in the synthesis.

Source group	Examples	Synthesis role	Principal limitation
Definitions and surveillance	CDC, UNESCO, WHO HBSC, National Academies	Operational definitions, burden estimates, cross-national context	Thresholds, recall periods, and survey frames differ.
Systematic reviews and meta-analyses	Prevalence, internalizing symptoms, suicidality, sleep, intervention	Highest-weight quantitative evidence and heterogeneity assessment	Primary-study quality and construct variation are inherited.
Longitudinal cohorts	Prospective mental-health, adult, educational, and biological outcomes	Temporal ordering and life-course interpretation	Residual confounding and attrition prevent simple causal claims.
Cross-sectional and nationally representative studies	Vulnerability, provider practice, population patterns	Population differences and clinically relevant correlates	Directionality cannot be established.
Intervention and implementation studies	Whole-school, cyberbullying, parenting, nursing, quality improvement	Effect estimates and healthcare feasibility	Effects are modest and heterogeneous; healthcare evidence is limited.
Measurement and clinical guidance	OBVQ-R, ECIP-Q, pediatric reviews and practice articles	Assessment domains and potential responses	No instrument or guideline identified validates BV-CARE or supports universal screening.

Note: The complete 58-source set is listed in the References; main-text Tables 1–6 cite the source groups used for each synthesis.

Table S3. SANRA self-assessment after revision.

SANRA item	Score	Basis
Justification of importance	2/2	Clinical burden and translational gap are explicitly established.
Statement of aims	2/2	Three research questions define construct, evidence, and healthcare objectives.
Description of literature search	1/2	Databases, dates, strings, eligibility, and final source count are reported; no prospective record log or duplicate screening.
Referencing	2/2	Claims and all tables are linked to numbered sources in order of first appearance.

SANRA item	Score	Basis
Scientific reasoning	2/2	Association, causation, bidirectionality, heterogeneity, and uncertainty are distinguished.
Presentation of relevant data	2/2	Prevalence, longitudinal, intervention, and limitation data are reported quantitatively where supportable.
Total	11/12	Conservative self-rating reflecting the non-systematic search and selection process.

SANRA = Scale for the Assessment of Narrative Review Articles [31].
